# Resilience or hope? Incremental and convergent validity of the resilience scale for adults (RSA) and the Herth hope scale (HHS) in the prediction of anxiety and depression

**DOI:** 10.1186/s40359-017-0205-0

**Published:** 2017-10-27

**Authors:** Roxanna Morote, Odin Hjemdal, Karolina Krysinska, Patricia Martinez Uribe, Jozef Corveleyn

**Affiliations:** 1 Department of Psychology, Norwegian University of Science and Technology, Trondheim, Norway; 2 Dementia Collaborative Research Centre, University of New South Wales, Sidney, Australia; 3 Department of Psychology, Catholic University of Peru, Lima, Peru; 4 Department of Psychology, University of Leuven, Leuven, Belgium

**Keywords:** Resilience, Hope, Anxiety, Depression, Incremental validity, SEM

## Abstract

**Background:**

Hope and resilience protect against inner vulnerabilities or harsh life circumstances; they explain individual differences in physical or mental health outcomes under high stress. They have been studied in complementary or competing theoretical frameworks; therefore, the study of measures of hope and resilience should be undertaken prior to explore if they are truly value-added for research. This study investigates the convergent and incremental validity of the Resilience Scale for Adults (RSA) and the Herth Hope Scale (HHS), in the prediction of anxiety and depression (HSCL-25).

**Methods:**

Participants in this community-based sample are 762 adults from 18 to 74 years old. They answered the RSA, HHS, Spanish Language Stressful Life-Events Checklist (SL-SLE), and the Hopkins Symptom Checklist-25 (HSCL-25). Incremental validity analyses combined hierarchical regression and structural equation models (SEM). First, hierarchical regression models were compared based on three criteria (*R* ^*2*^_*Diff.,*_ *ΔF*, and semi-partial *r*), then the direct effect of resilience on affective symptoms was compared with the mediated effect of resilience on affective symptoms through hope.

**Results:**

The hierarchical models showed that (1) hope and resilience account significantly for the variance of affective symptoms above age, sex, and life-stress; (2) Resilience Total score has greater incremental validity than positive scales of HHS Hope; and (3) RSA Total score, HHS Optimism/Spiritual support, Stressful life-events and sex are unique predictors of affective symptoms. The SEM analyses verified a stronger direct effect of resilience in the prediction of affective symptoms above the significant partial mediated effect of resilience through hope. Additionally, results show that age and better educational opportunities were associated with protection (i.e. resilience and hope) and emotional well-being (i.e. affective symptoms and hopelessness). Women showed higher scores in social competences and resources (RSA), interconnectedness and initiative to take action (HHS). However, they have poorer evaluations of own abilities and efficacy (RSA), and higher scores in all the affective symptoms assessed.

**Conclusion:**

The RSA has incremental validity above the HHS, however, both the RSA and the HHS are effective, differentiated and complementary measures of protection that are of high relevance for research on psychosocial and emotional well-being.

**Electronic supplementary material:**

The online version of this article (10.1186/s40359-017-0205-0) contains supplementary material, which is available to authorized users.

## Background

Resilience and hope are sources of inner strength that contribute to human development and well-being across the lifespan; they can also protect against the impact of negative life events and psychopathology [1, 2]. Resilience and hope have been studied either separately, as part of the same conceptual framework (i.e. hope as an aspect of resilience), or as moderating or mediating constructs in the development of negative outcomes [3–7]. However, in the recent and growing literature of health enhancing mechanisms, it is important to clarify the differences (at theory and measurement levels) between related constructs [8]. That is particularly needed if the contextual relevance of the new instruments is sought, for instance, in multicultural contexts where instruments have not been developed, or when research aims at representing multiple dimensions of local experiences with valid tools. In this study, we aimed to identify and to evaluate psychometric instruments of resilience and hope that may reflect this complexity and may be used in a complementary fashion to predict mental health.

Incremental validity analysis uncovers the relative contributions of different variables to some outcome variable. In hierarchical regression models, a set of variables are regressed on an outcome variable looking for those predictors that remain significant after controlling for the others in successive steps. In the last years, incremental validity techniques based on regressions have been criticized because of the lack of control of measurement error. Currently, Structural Equation Models (SEM), more precisely, mediation models, have been used successfully to control for measurement errors in incremental validity studies [9–11]. This study aims at investigating the incremental validity of two measures of protection, the Resilience Scale for Adults [12, 13] and the Herth Hope Scale [14, 15] in the prediction of affective symptoms combining hierarchical regression models and mediation analyses. The study also explores the associations of the scales with potential risk factors, such as life-stress, age, sex, and education.

In the following sections, we will deepen our understanding of adult resilience and hope in relation to mental health and well-being; we will also discuss the complementarity of the techniques of incremental validity used. The study will contribute to an empirical framework to investigate hope and resilience in a Spanish-speaking Latin American context.

### Resilience and hope: Protective aspects in adulthood

Research on protective mechanisms, such as resilience and hope, is a relatively new field in clinical, health and positive psychology. Researchers have approached them within different frameworks, focuses, and mainly in relation to positive or negative outcomes of health and well-being. Resilience has been studied as a trait, as a developmental process, as an outcome of adaptation. It has been depicted as a multi or one-dimensional construct, a pattern of recovery, and it has been studied in interaction or not with external adversities [16, 17]. Today, resilience research aims at integrating multiple levels of analysis, from gene-environment interactions to the complex process of adaptation in individual, family, peer, and community levels [18, 19]. As protective mechanisms, adult resilience allows some people to face back actual risks above conventional expectations, thus explaining individual differences in the processes of adaptation [16, 20].

The instrument developed by Friborg et al. [13] is one of the few valid methods to evaluate adult protective mechanisms. The Resilience Scale for Adults (RSA) was developed following inductive procedures: identification of protective factors in specialized literature, categorization, and empirical reduction of domains [13, 21]. The RSA evaluates four intrapersonal mechanisms of protection: confidence in abilities and judgments, and self-efficacy, the ability to plan ahead, being goal-oriented and having a positive outlook, the preference for having and following routines, and social warmth, flexibility and humour; as well as two social and family oriented mechanisms of protection [21, 22]. Research has demonstrated that RSA protective factors buffer the effect of stress thus preventing the development of affective symptoms, pain or general mental health issues [23–25].

Today, psychological perspectives of hope define it as a multidimensional.. Hope can be conceptualized as positive expectations about a possible and significant future good, either in a specific (time-limited) or global perspective [26, 27]. The Herth Hope Scale (HHS) was designed as a multi-facet instrument [14]. Based on the theoretical model of Dufault and Martocchio [28], the HHS evaluates aspects of particularized hope (time-valued outcome) as well as a generalized sense of transcendence and meaning [14]. Originally, the HHS was designed to measure three aspects of hope (i.e. cognitive, affective and affiliative/spiritual). However, recent studies in diverse cultural contexts suggest different internal structures [29, 30], and/or an independent sub-facet of hopelessness [31, 32]. In its Spanish-language version, the Herth Hope Scale evaluates aspects of optimism/spiritual support, hopelessness, belonging/social support, and agency [15].

In terms of contextual relevance, the domains and contents of both the above-mentioned instruments are relevant to investigate adult protective factors in Latin America. The RSA evaluates personal as well as family and socially oriented attributes, while the HHS adds transcendental, affiliative and motivational components of protection. Although still scarce [33], research in Latin America has shown that these elements must be considered to understand complex processes of overcoming adversity, building of communities or facing psychosocial risk or mental health challenges in multicultural contexts [34, 35].

### Resilience and hope in the search for mental health and well-being

Psychology and health-related research have demonstrated that mechanisms of resilience and hope impact on the well-established relation between life-stress and psychopathology symptoms [36, 37], as well as on health behaviors and indicators of physical health (i.e. cardiovascular function, immune system) [38]. A meta-analytic review confirmed the protective impact of resilience in relation to depression and anxiety, and secondly, in relation to post-traumatic stress disorder (PTSD) and negative affect [39]. Longitudinal studies have shown that multiple and constant family disadvantages [40], the quality of later relationships [41] and positive adult experiences against adversities [20] are at the base of individual differences in adult resilience.

Hope acts together with other inner resources as a protection against external threats or inner vulnerabilities. Recently, the mediation role of hope in the relationship of resilience and well-being was found in adolescents [6]. Clinical studies reported negative associations of hope and symptoms of depression, anxiety, and psychological distress, and conversely to adaptive coping, subjective and spiritual well-being, and immune response [27, 29]. Hope, along with optimism about the future and empowerment, have been pointed as core dimensions of the process of recovery from mental illness [42–44]. Hopefulness is a component of positive psychological well-being contributing to reduced all-cause mortality in healthy populations [45] and enhancing life quality and recovery in diverse medical conditions [46–48]. On the other hand, hopelessness has been clearly associated with anxiety or depression [49–51]. More recently, hopelessness has been studied in a depression-anxiety co-morbidity model [52]. Hopelessness is a recognized risk factor for both suicidal behavior and non-fatal deliberate self-harm [53–55].

### Incremental validity analysis

In clinical psychology, the question of added value in the combined use of two different instruments is rarely addressed although it has been claimed for decades [56]. The search for incremental value is of crucial importance to gain efficacious predictions and efficiency in mental health evaluations [57]. Recently, criticism has risen from the confirmation that most incremental validity studies reach their conclusions based on only one estimate (*β* values) of multiple hierarchical regressions models, and that researchers tend to generalize these conclusions to the latent variable level [9].Hierarchical regression models analyze if a specific variable predicts the variance of an outcome after controlling for the effect of other predictors in sequential steps. These steps are determined by previous research. In the past decade, researchers warned about inferring causal relations by controlling certain variables because confounders may remain covered or covariates may dilute other significant associations [58]. This is particularly relevant for incremental validity studies when instruments assessing associated constructs (e.g. protective factors) are tested. Due to these risks, researchers suggested that the combination of several criteria [59, 60] and the comparison of several hierarchical regression models [61] are better strategies to determine which measures matter in predicting over and above other measures.

However, hierarchical regression models use the Ordinary Least Squares (OLS) method whose assumptions are rarely met, thus increasing the risk of error type I (i.e. the incorrect rejection of a true null hypothesis or false positive error). In this context, just recently, researchers have shown the benefits of adding a Structural Equation Modelling approach (SEM) to the conventional regression approach in determining the incremental predictive value of associated measures. The SEM approach is a data analytic strategy that does not assume the absence of measurement error. The error is incorporated into the equation as a residual term associated with the observed variables, and therefore measurement level variables might be treated as latent variables. Mediation model has been used to test incremental validity hypotheses by comparing the direct effect of a predictor on an outcome variable with the effect of the predictor mediated through a third variable [6, 10].

Some authors have pointed towards the importance of differentiating salutogenic factors in mental health predictions via the SEM approach to allow new constructs to delineate their use and potential among theoretical or classical constructs [62]. The SEM approach should provide suitable evidence for construct-level incremental validity conclusions taking into account the possible pitfalls of measurement [9].

The combined and accurate measurement of protective factors associated with resilience and hope is relevant to build up a broad perspective of adults’ inner strengths and resources at empirical and theoretical levels. Moreover, it must incorporate the analysis of basic psychosocial conditions (such as age, sex, and educational attainment) that have proved to influence how individual mechanisms lead or not to positive adaptation and well-being [63, 64].

## Methods

This study aims at exploring the convergent and incremental value of two measures of protective mechanisms: The Resilience Scale for Adults (RSA) and the Herth Hope Scale (HHS) in relation to outcomes of psychopathology (anxiety and depression evaluated with the HSCL-25). The incremental validity analysis will combine two methods: hierarchical regression models and structural equation models. The analyses will take into account the control of relevant conditions such as life-stress (SL-SLE), age, sex, and education.

### Participants

The sampling process was non-probabilistic, convenient and community-based. We wanted to reach a group of participants with a broad range of age, a comparable number of men and women, and with diverse levels of education. Therefore, participants were recruited through work, educational and social institutions. Eight hundred and forty-four Peruvian adults were invited as volunteers and they were informed about their rights as participants (informed consent). The inclusion criteria were to be Peruvian, to be older than 18 years of age, and to have completed elementary education. The participants answered a paper-based survey composed by the Resilience Scale for Adults (RSA), the Herth Hope Scale (HHS), Spanish Language Stressful Life-Events Checklist (SL-SLE), and the Hopkins Symptom Checklist-25 (HSCL-25). Seven hundred and sixty-two participants correctly completed the survey (response rate 90.28%).

### Instruments

A pragmatic approach was undertaken to identify measurement instruments of resilience or ‘protective factors of resilience’ and hope. The databases used were Medline, Scopus, and PsychInfo. The search was from 1990 to the present. Once the most used instruments were identified, further searches were carried out to find original psychometric research in diverse cultural settings with an emphasis on multidimensional construct definitions. We consider the criteria of purpose, application, validity (internal construct validity and criterion-related validity), reliability (internal and temporal stability) and sensitivity [65] to verify the psychometric properties of the scales. We also revised systematic reviews or meta-analytic studies. For resilience scales, in accordance with Windle et al. [66], the Resilience Scale for Adults is the only multidimensional psychometric tool (i.e. assessing intrapersonal and interpersonal factors) with adequate psychometric properties and tested in multicultural contexts. For instruments of hope, we verified that the most used and solid scale of hope (the Snyder Hope Scale) focusses on agency and planning to achieve goals [67]. Therefore, we selected the Herth Hope Scale due to its multidimensionality, psychometric properties, consistent use across cultures, and its recent validation in Peru. The characteristics of both instruments are presented as here.

### Resilience scale for adults (RSA)

The RSA evaluates intrapersonal as well as interpersonal and family aspects of resilience: Perception of the Self, Planned Future, Social Competence, Family Cohesion, Social Resources, and Structured Style [21, 68]. The RSA is a self-report instrument (33 items) with a reliable semantic differential format (internal consistency and test-retest reliability) [24].

The validity of the RSA in different cultural settings has been tested, with clinical and community samples. The six-factor structure of the RSA has been confirmed in Italy, Lithuania, South Africa and Peru [69–72]. In Brazil and Belgium, the metric invariance and criteria-related validity (with affective symptoms) were also verified [22, 73]. In Peru, the RSA Total Score and five RSA factors had good internal consistency (Cronbach’s α RSA Total = .90, scales from .70 to .80), one factor shows weak internal consistency (Structured Style) [72]. The RSA has significant negative associations with anxiety, depression, and hopelessness [74, 75].

### Herth hope scale (HHS)

The Herth Hope Scale (HHS) evaluates cognitive, affective, interpersonal and spiritual aspects of hope. It is a reliable and theory-driven instrument developed to evaluate three components of hope (i.e. Temporality and future; Positive readiness and expectancy and Interconnectedness) in healthy and ill adults [14, 76]. Meaningful associations of the HHS scores with relevant constructs have been found in North America [77–79] and Iran [80]. In Peru, the four components structure of the HHS was confirmed; good internal consistency was reported for the total scale (*α* = .90) and four scales: Optimism/Spiritual Support (*α* = .82), Hopelessness (*α* = .79), Agency (*α* = .78), Social-Support/Belonging (*α =* .736) [31]. In a group of college students, there were found positive associations of Hope with Sense of Coherence and Life satisfaction [15].

### The Hopkins symptom checklist (HSCL-25)

The HSCL-25 evaluates symptoms of anxiety and depression. Item responses range from ‘not at all’ (1) to ‘extremely’ (4); higher scores represent the intensification of symptoms [81]. In Peru, a confirmatory factor analysis (CFA) verified the two factor structure of the HSCL – 25 and its reliability (Total score, α = .90; Anxiety, α = .81; and Depression, α = .86) [82].

### Spanish-language stressful life events checklist (SL-SLE)

The SL-SLE evaluates a number of adult stressful-life events experienced throughout life. The total score may range from 0 to 20 life events. The instrument includes relevant events such as “changing economic status”, "being a victim of crime (assault, rape)", “death of close family member”, “surviving a disaster” or “family violence”. The increase of the SL-SLE total score is associated with the increase of anxiety, depression, and general distress. In Peru, participants reported 0 to 8 (Median = 3) stressful life-events [82].

### Data analysis

All statistical analyses were completed with IBM SPSS and AMOS Graphics (version 23). To handle the missing information, first, following the recommendations of the RSA developers, four participants with more than 10% missing responses in the RSA protocol were removed. We used the Little’s Missing Completely at Random (MCAR) Test to verify that the missing responses (one to three items) in seventy-three RSA protocols were completely at random (Chi-Square statistic = 1414.016, DF = 1133, Sig. = .629). The missing responses were replaced with the mean score for the subscale that the item belonged to. Then, we eliminated the participants with three or more missing responses (10% of the total number of items) in the HSCL-25 (thirty-three participants) and the HHS protocols (forty-five participants). The mean score of the item replaced the missing response in protocols with one or two missing responses (in fourteen HSCL-25 protocols, and ten HHS protocols). Before the imputations, we verified a good internal reliability per scale (Cronbach’s α > .70). The total number of participants with complete protocols was seven hundred and sixty-two. A file containing the data analyzed in this study is available (see Additional file 1).

Inter-scales correlations and conditions for the regression analyses were explored. Homoscedasticity was verified with the robust test of Levene with rank transformations for unequal and non-parametric samples. That is, the special version of the Levene test verified that the variance of error terms are similar across the predictors or independent variables (i.e. hope and resilience scales), thus allowing the development and testing of hierarchical regression models [83]. T-test and the corrected effect size estimate Hedges’ g were used for mean comparisons. An effect size (i.e. the size of the difference between two groups) larger than .02 will be interpreted as a significant medium effect for mean comparisons (e.g. an effect size of .35 means that the score of the average person in a group is .35 standard deviations above the average person in the other group).

A set of hierarchical models investigated the incremental validity of resilience (RSA) and hope (HHS) in the prediction of anxiety, depression and total distress (HSCL-25). The hierarchical models explored the relative proportion of variance in the dependent variables (affective symptoms) associated with the compared components of protection, resilience and hope (steps 3 and 4) above sex, age (step 1), and stressful life-events (step 2) [59, 60]. RSA Total Score and the positive scales of HHS were introduced in the third and fourth step of three hierarchical models (one for each dependent variable); then this order was reversed to determine which variable influence more the variance of the dependent variables when controlling for the other. The comparison followed three parameters: Adjusted *R* ^*2*^_*Diff.*_ (the difference of the increment between steps three and four), the *F* test of the robustness of the increment of each step [60], and semi-partial *r* of .15 to .20 as a reasonable contribution for the third and fourth step of the hierarchical models [59].

As a final regression analysis, after verifying that RSA total score has a greater incremental validity over HHS scales (i.e. RSA in step 4 and HHS in step 3), we tested the incremental value of each RSA factors (step four: RSA scales compared) above the three scales of HHS hope. This final model was used to compare the unique predictive capacity of each scale of the instruments (*β* weights) [61].

Finally, a SEM-based statistical approach was introduced [9]. Two structural equation models (SEM) were compared to demonstrate the strength of the direct effect of resilience on affective symptoms above a model that includes hope as a mediator of this relationship. The recommended method of MacKinnon, Fairchild, and Fritz [84] was used to verify the significance of the mediation.

## Results

The participants come from a convenience sample. They are 762 Peruvian adults living in Lima. Men are 40.6% (*n* = 306) and women are 59.4% (*n* = 448) of the total group. Participants’ age ranges from 18 to 74 years old (X = 28.54, SD = 10.48). They have undergraduate education (*n* = 466, 62.3%), postgraduate education (*n* = 214, 28.6%), and secondary or technical education (*n* = 68, 9.1%). Table 1 shows the means, standard deviations and Pearson correlations of all the variables studied. All the inter-scales correlations (RSA, HHS, HSCL-25) have the expected direction and are significant at *p* < .001. Positive correlations between elements of protection (RSA, HHS), and negative correlations between them and emotional distress (affective symptoms and HHS hopelessness) confirm the convergent validity of the RSA and the HHS.

**Table 1:** Means, standard deviations and Pearson’s correlations between demographics, SL-SLE, RSA, HSCL, and HHS (*N* = 762)

		Mean	SD	1	2	3	4	5	6	7	8	9	10	11	12	13	14	15	16	17
1	Sex																			
2	Age	28.54	10.48	−.04																
3	Education			.04	.04															
4	SL- SLE	3.74	2.72	.03	**.42**	.02														
5	RSA Perception Self	5.37	1.03	−.10**	**.34**	.05	**.15**													
6	RSA Planned Future	5.06	1.09	.02	**.18**	.11**	.05	**.60**												
7	RSA Soc. Competence	5.31	.99	.08*	**.24**	.03	.12**	**.45**	**.38**											
8	RSA Fam. Cohesion	5.47	1.09	.02	**.24**	.00	.04	**.45**	**.40**	**.39**										
9	RSA Soc. Resources	5.99	.81	**.17**	**.14**	.05	.05	**.45**	**.42**	**.59**	**.54**									
10	RSA Structured Style	5.17	.10	−.01	**.25**	.03	.03	**.39**	**.41**	**.26**	**.31**	**.26**								
11	RSA Total	5.45	.72	.04	**.32**	.06	.10**	**.78**	**.71**	**.73**	**.74**	**.77**	**.56**							
12	HSCL Anxiety	1.50	.39	.12**	**−.18**	−.09*	.01	**−.55**	**−.39**	**−.30**	**−.31**	**−.34**	**−.24**	**−.50**						
13	HSCL Depression	1.49	.41	.08*	−.07	−.12**	.08*	**−.56**	**−.49**	**−.32**	**−.38**	**−.39**	**−.27**	**−.56**	**.71**					
14	HSCL Total	1.50	.37	.10**	−.12**	−.11**	.05	**−.59**	**−.48**	**−.34**	**−.38**	**−.40**	**−.28**	**−.57**	**.89**	**.96**				
15	HHS Optimism /Spiritual Support	3.50	.47	.05	**.24**	**.15**	.09*	**.62**	**.46**	**.41**	**.42**	**.45**	**.30**	**.62**	**−.39**	**−.48**	**−.47**			
16	HHS Hopelessness	1.95	.60	.06	−.10**	−.08*	.041	**−.52**	**−.49**	**−.27**	**−.32**	**−.33**	**−.28**	**−.51**	**.53**	**.62**	**.63**	**−.35**		
17	HHS Agency	3.71	.43	**.15**	.08*	**.18**	.037	**.42**	**.47**	**.30**	**.30**	**.39**	**.34**	**.50**	**−.24**	**−.37**	**−.35**	**.68**	**−.29**	
18	HHS Soc. Support/Belonging	3.57	.48	**.17**	.12**	.10**	.01	**.38**	**.37**	**.45**	**.46**	**.63**	**.22**	**.59**	**−.35**	**−.43**	**−.43**	**.61**	**−.32**	**.55**

Spanish-Language Stressful life events (SL-SLE), Resilience Scale for Adults (RSA), Hopkins Symptom Check List (HSCL-25), Herth Hope Scale (HHS). All scales are scored such that higher numbers represent higher levels of the constructs

Sex is a categorical variable (male = 0, female = 1) and education is ordinal (high school = 1 to postgraduate education = 3)

All correlations above > .14 are significant at *p* < .001 (two-tailed) and are **bold**

* *p* < .05 ** *p* < .01 (two-tailed)

All the scale scores are non-normally distributed (Shapiro-Wilk test *p* = .000; standardized skewness above +/− 3.67). Positive constructs (hope and resilience) are left-skewed, while HHS Hopelessness, affective symptoms (HSCL-25) and stressful life-events (SL-SLE) are right-skewed. Interestingly, the total score of stressful life-events is significantly positively associated with aspects of protection (RSA and HHS scales) as well as with the increased depression (HSCL-25). Age is positively significantly associated with all the aspects of protection (RSA, HHS) and negatively associated with HHS Hopelessness, anxiety and HSCL-25 total score. Education is significantly associated with all HHS scales: positive dimensions of hope increase with higher levels of education (positive correlations) while Hopelessness decreases (negative correlation). Education is positively associated with one RSA scale: Planned Future.

Consistent with the literature [40, 85], sex is significantly correlated with specific aspects of protection. Female gender is significantly associated with resources such as RSA Social Competence, RSA Social Resources, HHS Social Support/Belonging, and with HHS Agency (an initiative to take action). However, women also experience higher levels of affective symptoms (HSCL Anxiety and Depression) as well as lower levels of RSA Perception of the Self.

In order to verify these sex differences, the means of all the scale scores were compared by sex. Table 2 shows significant differences in variance and score means by sex. Women score higher on RSA Social Competences and Social Resources, HHS Agency and Spiritual Support/Belonging, as well as on affective symptoms: HSCL Anxiety, Depression, and Total Score. RSA Perception of the Self is the only protective mechanism where men have higher mean scores.

**Table 2:** Significant sex differences for RSA, HSCL and HSS mean scores (n = 762)

Scales	Levene Test Ranks		t – test		Men (*n* = 320)		Women (*n* = 477)		Hedge’s g
	*F*	*p*	*T*	*p*	*M*	*SD*	*M*	*SD*	
RSA Perception of Self			2.40	*	5.47	1.00	5.29	1.06	.17
RSA Social Competence			−2.22	*	5.22	1.03	5.38	.97	.16
RSA Social Resources			−4.85	**	5.83	.86	6.11	.75	.35
HSCL Anxiety			−3.03	**	1.45	.38	1.53	.38	.22
HSCL Depression	4.73	*	−2.05	*	1.46	.40	1.52	.41	.15
HSCL Total			−2.62	**	1.45	.36	1.52	.37	.19
HHS Agency			−4.15	**	3.64	.49	3.77	.36	.30
HHS Belonging/Social Support	11.13	**	−4.63	**	3.48	.52	3.64	.42	.33

Resilience Scale for Adults (RSA), Hopkins Symptom Check List (HSCL-25), Herth Hope Scale (HHS)

*p < .05, ** *p* < .01 (2- tailed)

The size of the differences in RSA scores by sex is similar to those reported in other contexts [13]. For all the continuous latent attributes studied, significant sex differences are in expected margins and are not negligible (*t*-tests and Hedge’s *g*) considering that these are measures related to mental health in a broad community sample [86, 87].

### Incremental validity

The incremental validity analysis comprises a set of comparisons of hierarchical regression models and then the comparison of two structural equations models. Based on hierarchical regression analyses, Table 3 shows the incremental validity of the RSA (Total Score, step 4) above the positive components of hope (HHS, step 3) in the prediction of symptoms (HSCL- 25), after controlling for age, sex (step 1) and stressful life-events (SLE, step 2).

**Table 3:** Incremental validity of the RSA: hierarchical multiple regression models for variables predicting affective symptoms (*N* = 762)

	HSCL Total				HSCL Anxiety				HSCL Depression			
Predictors	*β*	*R* ^*2*^_*Adj.*_	*ΔR* ^*2*^	*ΔF*	*β*	*R* ^*2*^_*Adj.*_	*ΔR* ^*2*^	*ΔF*	*β*	*R* ^*2*^_*Adj.*_	*ΔR* ^*2*^	*ΔF*
Step 1: Demographics		.02		**9.32**		.04		**17.65**		.01		4.20*
Sex	**.11**				.11**				.09**			
Age	.04				−.04				.09**			
Step 2: SL- SLE		.04	.02	**13.71**		.06	.01	9.34**		.03	.02	**13.47**
Stressful life-events	**.12**				.10**				**.11**			
Step 3: HHS Hope		.26	.23	**74.40**		.19	.14	**42.05**		.26	.24	**78.17**
Optimism/Spiritual Support	−.04				.01				−.06			
Soc. Support/Belonging	**−.19**				**−.19**				**−.18**			
Agency	.07				.10				.05			
Step 4: RSA		.37	.11	**127.40**		.27	.08	**79.25**		.37	.11	**119.79**
Total Score	**−.46**				**−.40**				**−.45**			
Step 4: RSA scales compared		.42	.16	**34.45**		.32	.13	**23.02**		.41	.15	**31.17**
Perception of Self	**−.40**				**−.41**				**−.35**			
Planned Future	**−.14**				−.07				**−.17**			
Social Competence	−.06				−.06				−.06			
Family Cohesion	−.03				.01				−.04			
Social Resources	−.01				.00				−.01			
Structured Style	−.03				−.04				−.03			

Spanish-Language Stressful life events (SL-SLE), Resilience Scale for Adults (RSA), Hopkins Symptom Check List (HSCL-25), Herth Hope Scale (HHS). Sex: 0 = male; 1 = female, age is mean centred. Bonferroni adjusted alpha level for the models compared (2 × 3 independent variables) is .0083. Variance inflation factor (VIF) and Tolerance were in accepted levels. Significant estimates at *p* < .001and are **bold**

**p < .01; *p < .05

The exploration of the incremental validity (increment of *R* ^*2*^_*Adj.*_, the significance of *ΔF*, semi partial *r*) required the comparison of two hierarchical models for each dependent variable (HSCL Total, Anxiety and Depression). First, RSA Total score was added in the third step and HHS positive scales were added in the fourth step (comparison models, not in table). Then, the order of the variables in steps three and four was reversed and the estimates of incremental validity were explored. Table 3 summarises the results for the models accounting for the greater amount of variability of the outcome variables.

As expected, the total amount of variance explained in the dependent variables is the same in the models compared. The *ΔF* (F Change) of all the steps in the two groups of models compared (including models with HHS scales in the fourth step) are significant (mainly at *p* < .01). However, the increase of the prediction (Adjusted *R* ^*2*^_*Diff.*_) and strength of the semi-partial correlations (*r*) are higher when Resilience Total is added in the fourth step above Hope scales in the third step (Table 3).

In the comparison models, when Hope scales were introduced in the fourth step (after Resilience Total Score in the third step), the increase of *R* ^*2*^_*Adj.*_ of the model (*R* ^*2*^_*Adj.*_ Change) was significant. The values of the increase were *ΔR* ^*2*^ = .020 for HSCL Total score, *ΔR* ^*2*^ = .012 for Anxiety, and *ΔR* ^*2*^ = .023 for Depression. However, in those models, the semi partial correlations of each HHS scale did not reach the criteria suggested by Hunsley and Meyer [59] for increment in third and fourth steps (*r* > .15).

In contrast, when the order is reversed (Table 3), the increase of the prediction between steps three (Hope) and four (Resilience) is five to seven times bigger (Adjusted *R* ^*2*^_*Diff.*_). The explained variance of the dependent variables reached 37% for HSCL Total score, 27% for Anxiety and 37% for Depression (*R* ^*2*^_*Adj.*_) in the final step. RSA Total Score remains highly correlated to the dependent variables when added either in step three or four in the six models (semi partial *r* > .30). Therefore, although Hope scales are good predictors of affective symptoms (in step three), Resilience Total Score (in step four) has greater incremental validity in the prediction of affective symptoms above and beyond positive dimensions of Hope.

Table 3 summarises these results, thus *β* weights correspond to the step where the variables added for the first time. In the final models (when all variables were added in the fourth step) some independent variables remain significant unique predictors along with RSA Total score (*β* weights in the last step, at *p* < .001). Sex, Stressful life-events and HHS Optimism/Spiritual support are unique predictors for HSCL - Total score (*β* = .143, .103, −.164, respectively) and Depression (*β* = .125, .103, −.169, respectively); while only sex is a unique predictor for Anxiety (*β* = .146) together with RSA Total score (*β* = .156).

In an additional model, the RSA Total score was replaced by the six RSA factor scores in the fourth step (Table 3, Step 4: RSA scales compared), in order to compare the unique predictive capacity of the RSA factors. As a result, the increase of the prediction in step four of the model is notably larger (*ΔR* ^*2*^) than with the Total Score of the RSA in that final step. The models with the six RSA scales in the last step account for the highest percentage of variance of the dependent variables: 42% of HSCL Total score, 32% of Anxiety and 41% of Depression (*R* ^*2*^_*Adj.*_). Perception of the Self and Planned Future, remain good unique predictors (*β* weights) of HSCL Total Score and Depression while Perception of the Self is significant for Anxiety.

**Fig. 1:**
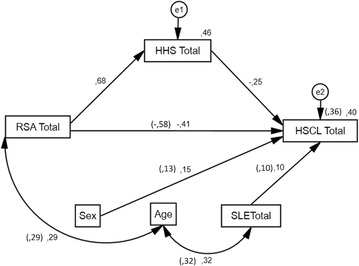
Direct effect and Mediation Models. Standardized regression coefficients and variance explained in HSCL. The estimates of the direct effect model are in parenthesis. Total scores of Resilience Scale for Adults (RSATotal), Hopkins Symptom Check List (HSCLTotal), Herth Hope Scale (HHSTotal), and Spanish-Language Stressful life events (SLETotal). Missing cases in age and sex decreased the sample size to *n* = 675. *p* < .001

Finally, the predictive relationship between protective mechanisms of resilience and hope, and mental health was tested in two structural equation models. The theory-driven hypothesis is that factors of protection have a negative effect on mental health. Based on the previous analysis, we aimed at demonstrating that (1) there is a negative direct effect of resilience (RSA total) on affective symptoms (HSCL25 total), and (2) the direct effect of resilience (RSA total) on affective symptoms (HSCL25 total) is stronger than the indirect effect of resilience on affective symptoms through hope (HHS total) (mediating partial effect). Three relevant control variables were included: stressful life-events (SLE total), sex and age. The mediation model is proposed in order to discard other possible relations between the variables of study (confounding, covariance or moderation) [10]. Figure 1 shows the standardized regression coefficients and variances of the mediation model and of the direct effect model (in parenthesis).

Figure 1 shows the models with estimated standardized path coefficients. The goodness-of-fit indices showed good models fit: for the direct effect model *χ* ^*2*^(5) = 9.00, *χ* ^*2*^ */df* = .5, *p* = .1 RMSEA = .03, CFI = .99, TLI = .98; and for the mediation model *χ* ^*2*^(8) = 15.49, *χ* ^*2*^ */df* = 0.52, *p* = .05, RMSEA = .04, CFI = .99, TLI = .98. The addition of the covariates sex, age and stressful life events (SLE total) did not alter the paths in the models or affect conclusions regarding incremental validity, on the contrary they strengthened the model fit indices. Non-significant regression coefficients and covariance were eliminated in both models.

The relationship between resilience (RSA total) and affective symptoms (HSCL total) is mediated by hope (HHS total). The standardized regression coefficient between RSA total and Hope total, and between Hope total and HSCL total are significant. The mediation effect was confirmed by assessing the statistical significance of the resilience to hope relation (path A), and then the hope to affective symptoms relation (path B). The estimate obtained for A x B was .090 with a *p* < .001, with Confidence Intervals (90%) of .059 to .128 (2000 bootstrap samples) [84].

As shown in Fig. 1, the direct effect of the RSA total score on the HSCL total score (−.58) is stronger than the effect of the RSA in the mediation model (−.41), although it remained statistically significant (*p* < .001). The variance explained in HSCL by RSA is 36% and only increments in four points in the mediation model. Thus, resilience (RSA) was found to possess a non-negligible amount of incremental predictive validity (i.e., a direct effect) as a predictor of affective symptoms (HSCL), above and beyond hope (HHS).

## Discussion

### Incremental validity of resilience and hope

To the best of our knowledge, this is the first incremental validity study of the self-report measures of protective aspects of adult resilience (RSA) and hope (HHS). Our study has accomplished the main goals of incremental validity analysis by combining the established criteria of hierarchical regression analyses and structural equation models [10, 59, 60]. In the hierarchical regression models, RSA total score and HHS factor scores are good predictors of psychopathology symptoms, and when combined, the proportion of variance explained in the outcome variables is notably higher. Then, when models are compared, the proportion of variance in the dependent variables associated with RSA Total Score in the last step is higher than when HHS scales are in the fourth step. Hunsley and Meyer [59] assert that incremental validity studies must demonstrate the value of adding a construct into a statistical equation to predict a criterion.

The significant partial effect found in the mediation model confirms the conceptual relations between resilience and hope (in the prediction of affective symptoms). When the mediation model is compared with the direct effect model, the unique relationship between resilience and affective symptoms prevails. This result suggests that higher levels of adult resilience are associated with lower levels of affective symptoms independently of the influence of positive aspects of hope.

The literature review shows that recent research is uncovering numerous and diverse kinds of protective mechanisms. Based on SEM analyses, Sense of Coherence has shown better incremental predictive validity in relation to substance abuse and mental health above well-established measures of personality (i.e. neuroticism, extraversion, and self-efficacy) [62]. Mediation models also confirmed that Emotional Intelligence is a stronger predictor of Life Satisfaction above positive and negative affect [10]. Our results show that two measures of resilience and hope may be used to get a better prediction of the underlying protective mechanisms that boost emotional well-being.

At a theoretical level, incremental validity studies of self-measures require the careful verification of items’ wording, domain frames, and the match specificity of each measure with the outcome variables [57, 61]. The RSA was developed in a cognitive framework with inductive procedures. The HHS is a theory-driven instrument whose internal structure has been tested empirically. Therefore, despite the different construction and validation processes of these instruments, they can complement each other because their domains and item contents are not overlapped. The results further confirmed that there is no empirical redundancy between the RSA and the HHS, and that their combined use for research in community adult samples is coherent and feasible.

Today, clinical and health psychology have demonstrated that positive constructs such as hope have clinical utility. However, Hjemdal et al. [8] have addressed the necessity of greater clarity on how to define and research on mechanisms related to personal or transcendental meaning. For instance, a recent study found that hope fully mediated the relationship between resilience and subjective well-being in a group of adolescents, thus suggesting the relevance of hope in this stage of development [6]. There is a promising future in health related research by including positive constructs and going beyond conventional approaches of psychopathology or achievement.

In addition, the investigation of protective aspects of resilience and hope on adults’ well-being has been recently introduced to empiric studies in Latin America [33, 88]. Consequently, the study of conceptual similarities or differences between positive constructs, as well as the verification of the validity and efficacy of the combined use of instruments enriches a new field of research and intervention in a Latin America.

### Convergent validity and contextual relevance of factors of protection

In different contexts, demographic characteristics (i.e., age and sex) have been found to be significantly associated with resilience [39] and affective symptoms [89]. In the present study, age, education, and sex have distinctive and relevant relations with the protective aspects of hope and resilience, vulnerabilities or life-stress. Young Peruvian adults consistently show higher risk of developing affective symptoms (mainly, anxiety) or experiencing hopelessness. Aging is correlated to the development of a broad set of protective factors, either six dimensions of resilience or three positive dimensions of hope. Although it is still understudied, adult resilience is a developmental route explained in terms of specific emotional or cognitive elements [90] and positive process of adaptation along life [63, 91].

Unlike age, education is significantly related to the three positive aspects of HHS hope, one of resilience, and the three scales of affective symptoms. The positive expectations, sense of belonging and capacitive to take initiative outlined in the HHS scales as well as the cognitive capacity to plan ahead and being goal oriented (RSA Planned Future) are connected with higher levels of education in the Peruvian sample. The characteristics of the context might enlighten these results. Despite the recent economic growth, Latin-America remains as the continent of socioeconomic inequality [92], including educational inequity [93]. However, along decades, education has been positively valued as a personal, family and social investment that would guarantee access to rights and opportunities [94, 95]. Results confirm that the capacity to define goals and arrange a step-by-step strategy for accomplishing them with a positive outlook, is related to better educational achievement, as well as to emotional well-being, in Peruvian adults.

Four up to nine factors of protection are in favor of women. In the social sphere, women not only show better Competence and Resources (RSA factors) but also they express more interconnectedness and emotional involvement with others (HHS Belonging/Social support), as well as motivation to take action (HHS Agency). Interestingly, the support and connection with others are not limited to family members, as it has been commonly described in Latin America [18, 96]. As evaluated by the HHS, female participants’ social orientation comprises transcendental and social dimensions. Moreover, similarly to studies in different contexts [4], the initiative to take action is stronger in women than in men.

However, consistently with the literature [97], the increase of symptoms (HSCL Total Score, Anxiety, and Depression) and lower confidence in own abilities, judgments, and efficacy (RSA Perception of Self) are characteristics of female participants. In the Peruvian sample, women are not hopeless, they have more social aspects of protection as well as initiate action to face life circumstances, although they face important challenges in their personal appraisal and perceived emotional well-being.

### Limitations and further research

Firstly, our study has the limitations of a cross sectional research design. The data was collected in a specific point-time without manipulation of the information, thus we have not prior or posterior information that might confirm or reject our results. Therefore, we do not draw conclusions about causal relations between the variables of study.

Secondly, as described, our sampling method was non-probabilistic and convenient. Here, a potential source of bias is the detection of participants due to researchers’ bias (e.g. in choosing the institutions where the volunteers were recruited), and participants’ self-selection. Therefore, we do not extend our results to a population level (i.e. adults with complete elementary education living in the city of Lima).

On the other hand, convenience sampling has commonly low response rate in community-based studies. Although we have not found comparable data (i.e. community convenience sampling of Peruvian adults with 56 years of age range), we can assert that we obtained a high response rate and a sample size large enough to analyze incremental validity hypotheses. This might reflect the motivation of the participants and the appropriateness of the surveys used. The fulfillment of the assumptions allowing the regression and structural models analyses may reflect a good quality of the responses obtained. Besides, self-report measures of positive constructs such as resilience and hope may elicit socially desirable responses. In order to minimize this risk, participants were blinded to the study hypothesis (i.e. the expected relations between stress, mental health outcomes, and protective mechanisms).

As discussed in the introduction, statistical models based on linear regressions have the disadvantages of error measurement and hidden confounders. The use of mediation models to test incremental validity allows certain control of the measurement errors but they are not equivalent to the hierarchical regression models. Based on the literature, a strategy to minimize the effect of possible confounders (i.e. age, sex, life stress) was to include them in the models tested.

Ultimately, we emphasize that our results must be interpreted within the context of a relatively new area of research, which has not antecedents in Latin America. Further research must test the predictive and causal relations among the variables studied in prospective and controlled studies. Grevenstein et al. [62] suggest that longitudinal comparison of incremental validity contributes to the understanding of the relative importance and mutual relationships of health-related constructs. Carefully designed experimental studies should enhance the control for imperfect measures, possible confounders, or spurious covariates [58]. Studies including mental health measures (such as the HSCL) may include clinical samples in order to add important value to the results and possible uses of the instruments in clinical settings.

Notwithstanding these limitations, our results will contribute to the inclusion of mechanisms of protection (resilience and hope) in community-based research. The study of psychological resources is a key element to enhance the promotion and prevention of mental health and well-being, particularly in vulnerable groups such as women and young adults.

## Conclusion

Resilience and hope are relevant and complementary aspects of protection in adult well-being. The results verified the expected and positive associations between them and their negative association with vulnerabilities (affective symptoms and hopelessness) (convergent validity). The analyses demonstrated that resilience and hope may work separately or together to prevent the onset of psychological symptoms. Moreover, the Resilience Scale of Adults (RSA) has larger incremental predictive validity above the Herth Hope Scale (HHS). In addition, results demonstrated the relations of protection and vulnerabilities with risks, either as stressful life-events or psychosocial conditions (i.e. sex, age, and education), contributing to the contextual validity of the study. In conclusion, the complementary use of the Resilience Scale of Adults and the Herth Hope Scale adds value to the study of protective factors in relation to mental health outcomes in community samples.

## Additional file

Additional file 1: The file has the complete information of all the variables studied. The names and description of the eighteen variables. (CSV 105 kb)
